# Retinal and choriocapillaris perfusion are associated with ankle-brachial-pressure-index and Fontaine stage in peripheral arterial disease

**DOI:** 10.1038/s41598-021-90900-5

**Published:** 2021-06-01

**Authors:** Maximilian W. M. Wintergerst, Peyman Falahat, Frank G. Holz, Christian Schaefer, Robert P. Finger, Nadjib Schahab

**Affiliations:** 1 Department of Ophthalmology, University Hospital Bonn, Ernst-Abbe-Straße 2, 53127 Bonn, Germany; 2 Department of Internal Medicine II, Heart Center Bonn, University Hospital Bonn, Bonn, Germany

**Keywords:** Biomarkers, Cardiovascular diseases, Vascular diseases, Peripheral vascular disease, Biomarkers, Diagnostic markers, Eye diseases, Cardiovascular diseases

## Abstract

The purpose of this prospective case–control study was to assess whether parameters of retinal and choriocapillaris perfusion are altered in patients with peripheral arterial disease (PAD). Patients with PAD and healthy controls were imaged with swept-source optical coherence tomography angiography (OCT-A). Macula centered 3 × 3 mm OCT-A scans were acquired, binarized and perfusion was evaluated for vessel density (VD) and choriocapillaris non-perfused area. Clinical examination and non-invasive assessment included Fontaine staging, ankle-brachial-pressure-index (ABI) and vascular color-coded Doppler sonography. Fifty-two patients with PAD and 23 healthy controls were included. Superficial retinal VD was reduced in patients compared to controls (difference =  − 0.013, *p* = 0.02), decreased with higher Fontaine stage (*p* = 0.01) and correlated with ABI (*r* = 0.42, *p* < 0.0001, 95% confidence interval [CI] 0.23–0.58). Choriocapillaris non-perfused area was larger in patients compared to controls (difference = 3.64%, *p* = 0.002, 95% CI 1.38–5.90%) and significantly correlated with ABI (*r* =  − 0.22, *p* = 0.03, 95% CI − 0.40– − 0.03). Multivariate multiple regression analysis revealed a significant association of all OCT-A parameters with ABI and of deep retinal vessel density and choriocapillaris non-perfused area with Fontaine stage. In this first study of retinal and choroidal perfusion in patients with PAD we found both retinal and choroidal perfusion to be significantly impaired. OCT-A parameters could aid as indirect imaging biomarkers for non-invasive PAD staging and monitoring.

## Introduction

Over 200 million people worldwide are affected by peripheral arterial disease (PAD), which causes excess morbidity by cardiovascular events like stroke and myocardial infarction^1,2^. Patients with cardinal symptoms like intermittent claudication have a 2.6 higher 10-year-mortality and even asymptomatic patients’ mortality risk is twice as high compared with unaffected age-matched peers^3,4^. The only available causal therapy is addressing risk factors like smoking, hypercholesterolemia or hypertension, which is why early PAD diagnosis is important^5,6^. Current diagnostic imaging techniques include color-coded Doppler sonography, magnet resonance angiography, computed tomography angiography and catheter based intra-arterial digital subtraction angiography^7^. However, their resolution is limited to non-capillary vessels, they are invasive and have a number of additional limitations^8^. Thus there is a lack of non-invasive, reliable and objective biomarkers for monitoring of disease progression, both in the clinical setting, as well as for use in clinical studies.

Ocular biomarkers, largely based on measurements of retinal vasculature, have previously been shown to allow for cardiovascular risk stratification and prediction of risk for strokes and heart failure^9–14^. Recently retinal microvascular abnormalities on color fundus photographs were shown to be strongly associated with the incidence of PAD^15^. However, resolution of fundus photography is low and it is inherently limited as it is a two-dimensional imaging modality imaging a three-dimensional vascular network.

Optical coherence tomography angiography (OCT-A) is a reliable, high-resolution, non-invasive and three-dimensional imaging modality based on optical interference which enables microvascular visualization stratified for specific retinal and choroidal layers^16,17^. OCT-A images are generated by repeated scans of the same structure detecting motion, i.e. blood flow. A previous study using OCT-A in acute coronary syndrome found both perfusion density and vessel density to be decreased as well as a correlation of these ocular vascular parameters to cardiovascular risk scores^12^. However, so far no studies have assessed the value of ocular OCT-A as an indirect imaging biomarker in PAD.

In this study we used OCT-A to assess ocular microvascular involvement in PAD as it might provide an easily obtainable and reproducible indirect PAD imaging biomarker in the future.

## Methods

### Subject recruitment

In this exploratory case–control study we recruited patients with PAD from the outpatient-clinic at the Department of Angiology and healthy age-matched controls at the outpatient-clinic of the Department of Ophthalmology at the University Hospital Bonn, Germany. Approval was obtained from the ethics committee of the University Hospital Bonn (approval ID 047/18) and informed consent was obtained from all study participants. The study was conducted according to the tenets of the Declaration of Helsinki. Inclusion criteria were a diagnosis of PAD based on clinical staging according to Fontaine and characteristic changes of the arteries of the lower extremities in color-coded Doppler sonography or computer tomography angiography or pathologic ankle-brachial-pressure-index (ABI) according to the current classification of PAD^18^. We excluded all patients with diabetes mellitus (as this is known to have a profound effect on OCT-A parameters^19^) or any current ocular symptoms, a history of any ocular surgery (except cataract surgery) or a history of any ocular diseases, corneal, lens or vitreous opacities, poor image quality on OCT-A (signal-strength-index < 8 was excluded) or investigator-determined reduced image quality (in terms of image artifacts, distinguishability of the foveal avascular zone and vessel continuity). Every scan has been graded by two graders (MWMW and PF).

### Angiologic examination and past medical history

ABI (the ratio between the systolic pressure in the lower and upper extremities^20^ was measured with Doppler-technique as recommended from the American Heart Association of the dorsal pedal and the posterior tibial artery^21^. All measurements were performed with the use of appropriately sized pneumatic cuffs for both the ankle and the arm. The systolic ankle pressures were recorded with a handheld 5 MHz bi-directional pocket Doppler instrument by continuous wave velocity detection (Bidop ES-100V3, HADECO, Kawasaki, Japan). The lowest ABI from all extremities was used for further analysis, as it has a higher sensitivity for detection of PAD and for estimating prognosis^22,23^. ABI < 0.9 was classified as PAD with intima calcification and ABI > 1.3 as PAD with media calcification, given high positive predictive values of these thresholds^21,24^. Considering that the Fontaine stage can decrease by local (i.e. lower extremities) surgery, the worst ever Fontaine stage (FS; stage I: defined as asymptomatic or effort pain; stage IIA/IIB: pain starting over a walking distance of > 200 m / < 200 m; stage III: pain in rest; stage IV: trophic lesions in the lower extremities^18,25^) was determined by a standardized self-report of possible walking distance^26^. Participants also self-reported medical history including risk factors such as smoking, arterial hypertension, hypercholesterolemia, diabetes mellitus and history of cardiovascular events. In all control-participants, the peripheral pulses of the lower extremities were detectable. Any reported PAD symptoms were an exclusion criteria for the controls.

### Ocular image acquisition and analyses

Optical coherence tomography is based on back-reflected light and generates structural images of the different retinal and choroidal layers. OCT-A has been developed for a no-injection, dye-free method for visualization of retinal and choroidal perfusion^16^. This technique detects blood flow from static tissue by analyzing the change in optical coherence tomography signal caused by the blood cells’ motion (Doppler shift and speckle variance/decorrelation) and allows for high-resolution visualization of perfusion stratified for different anatomical retinal and choroidal layers^16^. We imaged the study participants with swept-source OCT-A with 100.000 A-Scans/second (Zeiss PLEX Elite 9000; Carl Zeiss Meditec, Dublin, California, USA). A 3 × 3 mm scan of the macula was performed. Every 3 × 3 mm scan consisted of 300 A-scans per B-scan and four repetitions at 300 B-scans. Every A-line was acquired over a depth of 3 mm and contained 300 × 300 pixel (10 µm/pixel). OCT-A images were generated using an optical micro-angiography (OMAG) algorithm. Using the proprietary algorithm from the Zeiss OCT-A system, we analyzed the superficial (spanning from the inner limiting membrane to the inner plexiform layer) and the deep (spanning from the inner plexiform layer to the outer plexiform layer) retinal layers and the choriocapillaris (spanning from 29 to 49 µm below the retinal pigment epithelium). Before analyses, the deep vascular layer and the choriocapillaris were automatically corrected for projection artefacts by the proprietary software provided by the OCT-A device. Additionally we imaged all participants with conventional optical coherence tomography (Heidelberg Spectralis; Heidelberg Engineering, Heidelberg, Germany) to confirm the absence of any retinal pathologies. For quantitative analysis of the 3 × 3 mm macula-scans images were binarized and skeletonized with Fiji^27^ (an expanded version of ImageJ^28^) and analyzed for vessel density as previously described^29^. Exemplary unprocessed and binarized superficial retinal OCT-A images are provided in the supplemental figure. The choriocapillaris was evaluated for percentage of non-perfused choriocapillaris area as previously described^30^.

### Statistical analysis

Student’s t-test, Wilcoxon rank sum test, Kruskal–Wallis test and Fisher’s exact test were used for descriptive statistical analyses as indicated. Statistical analyses were performed with R (R: A Language and Environment for Statistical Computing, R Core Team, R Foundation for Statistical Computing, Vienna, Austria, v4.0.3, 2020). Differences were considered significant if they exceeded the 95% confidence level. Eyes with a maximum Fontaine stage > I were pooled in one category to increase statistical power. For univariate and multivariate regression analysis we used linear mixed models including a random intercept for each patient.

## Results

### Demographics and clinical characteristics

Ninety-seven eyes from 52 patients with PAD and 34 eyes from 23 healthy controls were included. Characteristics of the sample are shown in Table 1. 51 eyes (53% of the PAD group) were from patients with systemic media calcification and 51 eyes (53% of the PAD group) from patients with cerebrovascular disease. Ten eyes were from patients with a history of stroke (10% of the PAD group) and 25 (26% of the PAD group) from patients with a history of acute coronary syndrome.

**Table 1:** Characteristics of the cohort.

	Mean (range) ± SD or n (%)		*p*-value
	Peripheral arterial disease	Controls	
Age	67.94 ± 9.47 (46–89)	69.26 ± 10.31 (46–83)	0.33
Sex (male)	69 (71%)	16 (47%)	0.02
Best corrected visual acuity (LogMAR + rounded Snellen equivalent)	0.05 ± 0.07 (20/20)	0.03 ± 0.06 (20/20)	0.07
Central retinal thickness (μm)	279 ± 24.03	276 ± 20.10	0.91
**Maximum Fontaine stage**			
I	59 (61%)	0	
IIa	8 (8%)	0	
IIb	26 (27%)	0	
III	4 (4%)	0	
IV	0	0	
Lowest ankle-brachial-index	0.92 ± 0.32 (0.16–1.49)	–	
OCT-A image quality (signal strength index)	9.57 ± 0.54 (8–10)	9.50 ± 0.66 (8–10)	0.79
History of smoking	31 (32%)	6 (18%)	0.13

SD = standard deviation; OCT-A = optical coherence tomography angiogaphy; Student’s t-test, Wilcoxon rank sum test and Fisher’s exact test were used for statistical analysis.

### Quantitative analysis of OCT-A images

**Figure 1:**
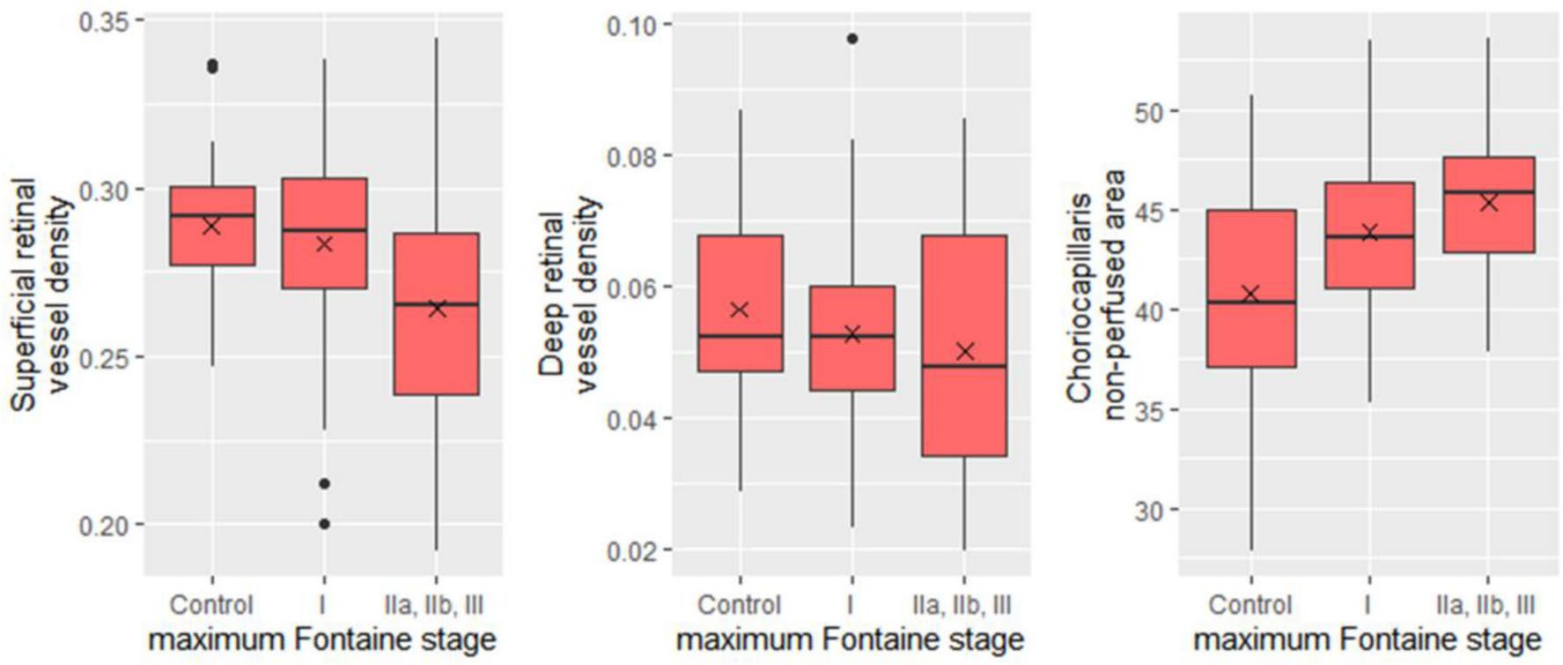
Decrease of retinal and choriocapillaris perfusion with higher Fontaine stage. Superficial and deep retinal vessel density and choriocapillaris non-perfused area on OCT-A were plotted against maximum Fontaine stage. Fontaine stages IIa, IIb and III were grouped.

**Figure 2:**
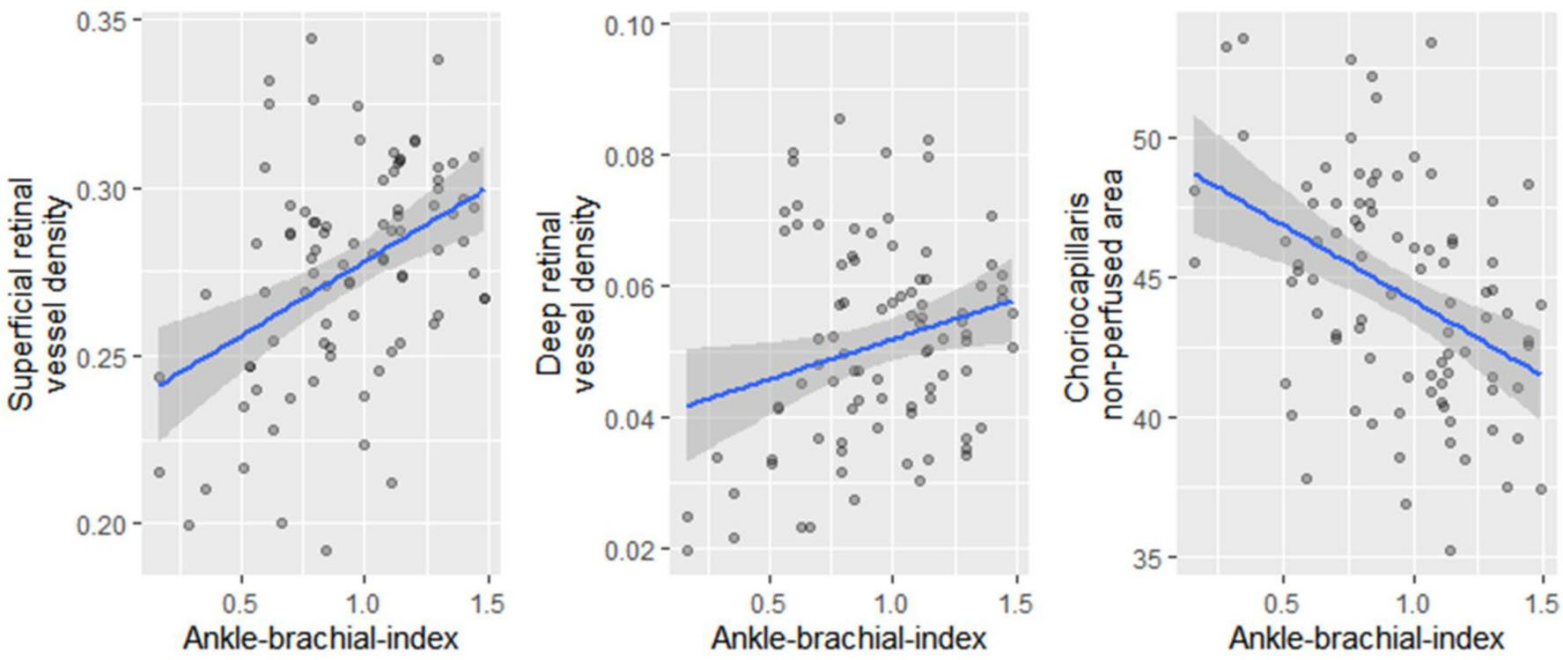
Correlation of retinal and choriocapillaris perfusion with ankle-brachial-index. Vessel density on OCT-A in the superficial and deep retina and choriocapillaris non-perfused area were plotted against ankle-brachial-index. The blue line represents a linear regression model with the surrounding confidence interval in light grey.

Superficial retinal layer vessel density and choriocapillaris non-perfused area were significantly associated with Fontaine stage (Fig. 1, Table 2) and there was a significant correlation of all OCT-A parameters with ABI (Fig. 2, Table 2). For the deep retinal layer, there was no significant difference for vessel density and no association with Fontaine stage (Fig. 1, Table 2). Post-hoc analysis revealed significant differences for superficial retinal vessel density between controls and pooled Fontaine stages IIa, IIb, III (*p* = 0.0016) and Fontaine stage I and pooled Fontaine stages IIa, IIb, III group (*p* = 0.0051) and for choriocapillaris non-perfused area between controls and Fontaine stage I (*p* = 0.017) and controls and pooled Fontaine stages IIa, IIb, III (*p* = 0.00047).

**Table 2:** Analysis of OCT-A parameters.

		Association with max. Fontaine stage^a^	Correlation with ABI^b^			Comparison of eyes from healthy controls and patients with PAD^c^			
OCT-A parameter		*p*	*r*	CI	*p*	Controls (Mean ± SD)	PAD (Mean ± SD)	Difference [CI]	*p*
Vessel density	Superficial retina	**0.0032**	0.42	0.23–0.58	** < 0.0001**	0.289 ± 0.022	0.276 ± 0.033	− 0.013 [− 0.002– − 0.024]	**0.021**
	Deep retina	0.29	0.25	0.056–0.43	**0.012**	0.056 ± 0.014	0.052 ± 0.016	− 0.0047 [0.0017– − 0.0110]	0.14
Non-perfused choriocapillaris area		**0.0019**	− 0.22	− 0.40– − 0.028	**0.026**	40.78 ± 5.44%	44.42 ± 4.12%	3.64 [1.38–5.90]	**0.0024**

^a^Kruskal–Wallis test; ^b^Pearson correlation; ^c^t-test / Wilcoxon rank sum test; ABI = ankle-brachial-pressure-index; CI = confidence interval; OCT-A = optical coherence tomography angiography; PAD = peripheral arterial disease.

### Subgroup analysis excluding eyes from patients with media calcification

As intima and media calcification are thought to be two distinct pathologies^24^, as the validity of the ABI for diagnosing PAD is reduced when there is media calcification^31^ and as media calcification has probably no effect on capillary perfusion, we excluded eyes from patients with media calcification in a subgroup analysis (resulting in 40 eyes for analysis). There was a larger significant overall difference in superficial retinal layer vessel density between controls and eyes from patients with PAD (mean [control group] = 0.289 ± 0.022, mean [PAD group] = 0.270 ± 0.035, difference of the mean = 0.019, t-test *p* = 0.0086, 95% confidence interval = 0.0050–0.0330) and choriocapillaris perfusion was more severely impaired than in the analysis of the overall cohort (mean [control group] = 40.78 ± 5.44, mean [PAD group] = 46.44 ± 4.19, difference of the mean =  − 5.66, t-test *p* < 0.0001, 95% confidence interval =  − 8.12 till − 3.20). There was no significant difference in retinal vessel density in the deep retinal layer (mean [control group] = 0.056 ± 0.014, mean [PAD group] = 0.051 ± 0.018, difference of the mean = 0.0052, t-test *p* = 0.19, 95% confidence interval =  − 0.0026 till 0.0130).

### Regression analysis

In multivariate multiple regression analysis using linear mixed models including a random intercept for each patient with the OCT-A parameters as dependent variables and age, OCT-A signal strength index, maximum Fontaine stage, ABI, sex, history of smoking, hypercholesterolemia, and arterial hypertension as independent variables, ABI and OCT-A signal strength index were associated with all OCT-A parameters, maximum Fontaine stage with deep retinal vessel density and choriocapillaris non-perfused area, arterial hypertension with choriocapillaris non-perfused area, and age with superficial retinal layer vessel density and choriocapillaris non-perfused area (Supplemental Table).

## Discussion

In this first study of retinal and choroidal perfusion in patients with PAD we found both retinal and choroidal perfusion to be significantly reduced. The results of this exploratory study imply an ocular asymptomatic involvement in PAD and retinal and choriocapillaris OCT-A parameters are potential candidates for non-invasive staging and monitoring of PAD and atherosclerosis in the future. This ocular asymptomatic involvement in PAD might be due to a common pathway with the general pathophysiology in PAD (that is atherosclerosis).

Associations of retinal perfusion with systemic cardiovascular disease have been described for acute coronary syndrome, stroke and heart failure^9–14^ and it has also been shown that PAD impacts microcirculation^32^. Furthermore, retinal microvascular findings on color fundus photographs (e.g. hemorrhages, microaneurysms, and exudates) can predict PAD progression in diabetes mellitus^33^, and it has been suggested that this also holds true beyond the potential confounding effect of diabetes mellitus^15^. As we excluded patients with diabetes mellitus in our study, our results support existing data that there is retinal microvascular involvement in PAD irrespective of presence of diabetes mellitus^33^.

Resolution of color fundus photography is inferior to OCT-A and stratification in different retinal layers and the choriocapillaris is impossible. In contrast, OCT-A allows to stratify between retinal and choriocapillaris perfusion and we also found microvascular abnormalities in the choriocapillaris of eyes from patients with PAD.

ABI was significantly associated with all OCT-A parameters and this association held true in multiple regression analyses. Interestingly, there also seems to be an association of ABI with optic nerve head circulation and development of diabetic retinopathy^34–36^. These findings might reflect a potential link of systemic atherosclerotic disease with ocular microcirculation. Furthermore, our results support existing literature on an association of arterial hypertension with impaired choriocapillaris perfusion^37,38^.

As intima and media calcification are thought to be two distinct pathologies^24^, as the validity of the ABI for diagnosing PAD is reduced when there is media calcification^31^ and as media calcification has probably no effect on capillary perfusion, we excluded patients with media calcification in a subgroup analysis. This revealed a greater difference in OCT-A perfusion parameters compared to the overall sample, which might reflect the different pathophysiological entities of media calcification and atherosclerotic intima calcification^31^. Hence, stratification of atherosclerotic intima and media calcification should be taken into account in future studies of microcirculation in atherosclerosis.

Our study demonstrates that OCT-A imaging biomarkers might be useful indirect measures for non-invasively staging and monitoring PAD. Although clinicians mainly rely on patient history and physical examination in guiding treatment, compared with the existing PAD outcome measures intra-arterial digital subtraction angiography and computer tomography angiography OCT-A is non-invasive, causes no radiation exposure and is less affected by intra- and inter-grader variation. Furthermore, currently used clinical measures such as ABI have several well-known limitations including low accuracy due to their subjective nature^39^. Against this, OCT-A offers an objective indirect parameter for PAD staging and monitoring which could aid in a study setting, e.g. for the development of novel interventions.

The strengths of our study are a comparison of OCT-A imaging biomarkers against the established clinical reference standards ABI and Fontaine stage, an objective automated image analysis approach, a reasonable sample size and stratification of our analysis in retinal and choriocapillaris perfusion. Our limitations include the cross-sectional nature of the study, therefore no causal relationships can be concluded. Furthermore, no assessment of ABI in the healthy controls has been done and the majority of our sample had only mild to moderate PAD. Another limitation is the different sex distribution between the control and patient group, as sex might have an effect on OCT-A parameters^40,41^. However, in our multivariate regression analysis sex was not significantly associated with any OCT-A parameter, so any present effect is likely very small. Owing to the cross-sectional design, our study cannot indicate whether observed changes are reversible or predictive of future disease progression and/or functional loss. As we did not investigate the effect of other reasons for atherosclerosis like diabetes and hypertension on the reported OCT-A parameters in our explorative study, our results cannot indicate the relationship. Furthermore, patients with diabetes are an important subpopulation in patients with PAD and can have falsely high ABI, which needs to be accounted for when using OCT-A for monitoring and staging PAD. Future, larger studies would benefit from including patients with diabetes and analyzing the confounding effect.

This is the first study assessing ocular microvascular perfusion in PAD, describing a previously unknown impairment and association of retinal and choroidal perfusion with ABI in PAD. OCT-A imaging parameters may aid as imaging-biomarkers for PAD staging and atherosclerosis monitoring. Further longitudinal studies on the prognostic value of OCT-A for cardiovascular disease and events are warranted.

## Supplementary Information

Supplementary Information 1.
