# Supplementary material for: Retinal and choriocapillaris perfusion are associated with ankle-brachial-pressure-index and Fontaine stage in peripheral arterial disease
Source: Sci Rep. 2021 Jun 1;11:11458. doi: 10.1038/s41598-021-90900-5 (PMC8169779; doi:10.1038/s41598-021-90900-5)
Supplement: Supplementary file 1 — Supplementary Information 1. [file 41598_2021_90900_MOESM1_ESM.docx]

**Retinal and Choriocapillaris Perfusion are associated with Ankle-Brachial-Pressure-Index and Fontaine stage in Peripheral Arterial Disease Maximilian W. M. Wintergerst^1,#^, M.D., Peyman Falahat^1,#^, Frank G. Holz^1^, M.D., Christian Schaefer^2^, M.D., Robert P. Finger^1,#,^*, Ph.D., Nadjib Schahab^2,#^, M.D.**

^#^ contributed equally, * corresponding author

^1^ Department of Ophthalmology, University of Bonn, Bonn, Germany

^2^ Department of Internal Medicine II, Heart Center Bonn, University Hospital Bonn, Bonn, Germany

**Author contribution statement:**

R.P.F., M.W.M.W., N.S. and C.S. conceived the study and provided the scientific overview with F.G.H.

P.F. recruited and imaged the participant and prepared the data for analyses.

M.W.M.W. performed the statistical analyses and generated the figures and tables.

M.W.M.W., P.F., R.P.F., N.S., C.S. and F.G.H. interpreted the Data.

The Introduction was drafted by P.F., M.W.M.W., R.P.F. and the Methods by P.F. and M.W.M.W. Results and Discussion were drafted by M.W.M.W.

All authors critically reviewed the manuscript and approved the final manuscript as submitted and agree to be accountable for all aspects of the work.

**Running title: OCT-A in Peripheral Arterial Disease**

**Corresponding author:**

Prof. Dr. med. Robert P. Finger, PhD

Chair of Ophthalmic Epidemiology and Neuroretinal Imaging

Department of Ophthalmology, University of Bonn

Ernst-Abbe-Straße 2 | 53127 Bonn, Germany; Email: robert.finger@ukbonn.de

Phone: 0049 228 287 15505 | Fax: 0049 228 287 14817


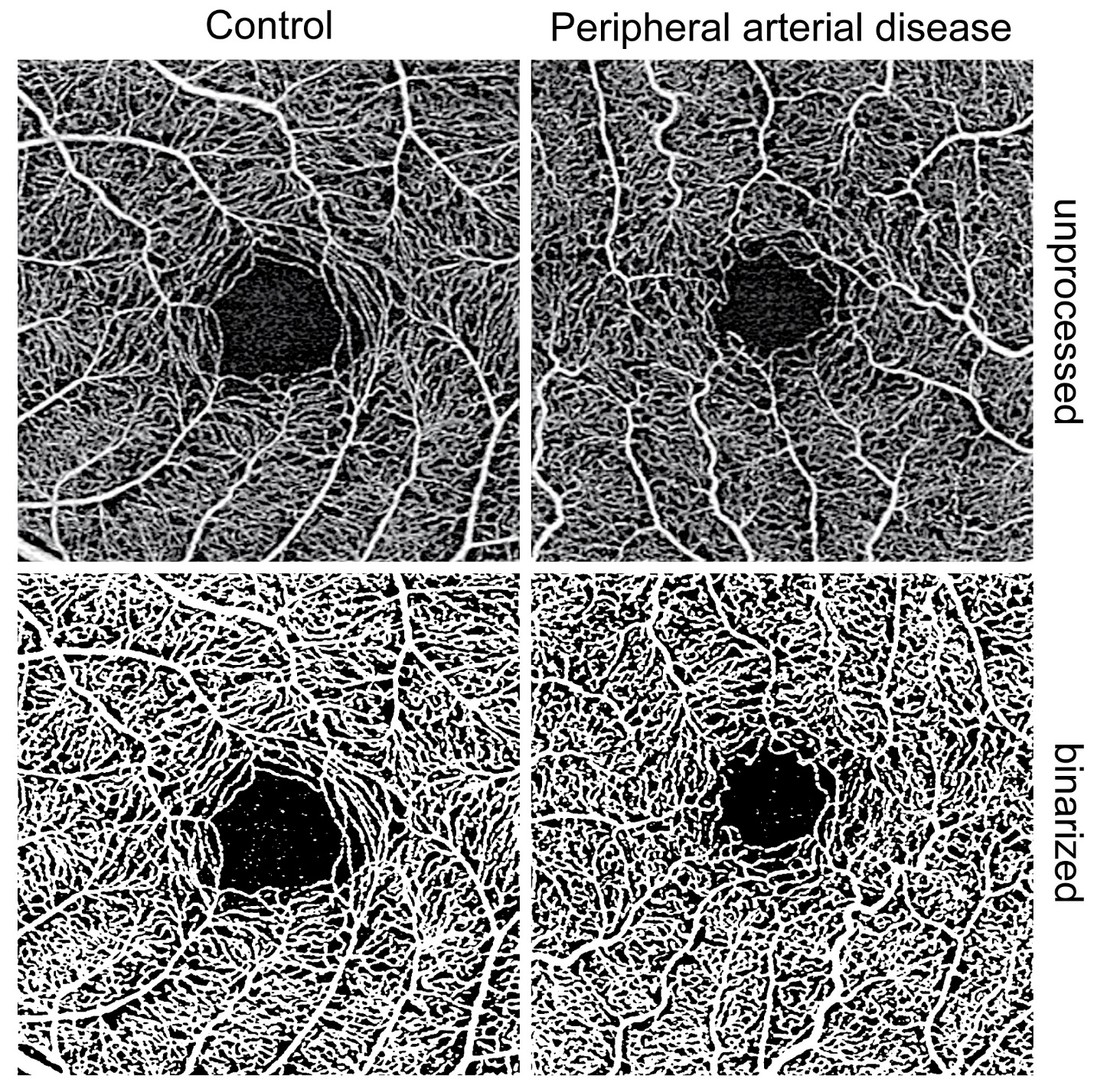


**Supplemental figure**. Exemplary unprocessed and binarized superficial retinal optical coherence tomography images

**Supplemental Table**. Multiple multivariate regression analysis using linear mixed models including a random intercept for each patient

|  | Superficial retinal vessel density | | | Deep retinal vessel density | | | Choriocapillaris non-perfused area | | |
| --- | --- | --- | --- | --- | --- | --- | --- | --- | --- |
|  | Estimate | Std. Error | p | Estimate | Std. Error | p | Estimate | Std. Error | p |
| Intercept | 9.6 x 10^-2^ | 6.2 x 10^-2^ | .13 | -6.1 x 10^-3^ | 3.1 x 10^-2^ | .84 | 6.7 x 10^1^ | 7.8 x 10^0^ | **< .0001** |
| Age | -8.9 x 10^-4^ | 4.4 x 10^-4^ | **.050** | -2.8 x 10^-4^ | 2.2 x 10^-4^ | .20 | 1.1 x 10^-1^ | 4.9 x 10^-2^ | **.034** |
| Sex | 8.5 x 10^-3^ | 8.4 x 10^-3^ | 0.32 | -4.0 x 10^-3^ | 4.2 x 10^-3^ | .34 | 4.4 x 10^-1^ | 9.4 x 10^-1^ | .64 |
| OCT-A signal strength index | 2.1 x 10^-2^ | 5.0 x 10^-3^ | **< .0001** | 5.5 x 10^-3^ | 2.4 x 10^-3^ | **.025** | -2.0 x 10^0^ | 6.8 x 10^-1^ | **.0039** |
| max. Fontaine stage | 6.5 x 10^-3^ | 1.2 x 10^-2^ | .59 | 1.2 x 10^-2^ | 5.9 x 10^-3^ | .**046** | -3.7 x 10^0^ | 1.3 x 10^0^ | **.0081** |
| ABI | 3.9 x 10^-2^ | 1.7 x 10^-2^ | **.026** | 2.2 x 10^-2^ | 8.4 x 10^-3^ | **.013** | -8.4 x 10^0^ | 1.9 x 10^0^ | **< .0001** |
| History of  smoking | -1.1 x 10^-2^ | 9.3 x 10^-3^ | .26 | 2.1 x 10^-3^ | 4.6 x 10^-3^ | .65 | 1.8 x 10^-1^ | 1.0 x 10^0^ | .86 |
| Hyper- cholesterolemia | -3.0 x 10^-3^ | 1.2 x 10^-2^ | .78 | -6.9 x 10^-3^ | 5.5 x 10^-3^ | .21 | -1.0 x 10^0^ | 1.2 x 10^0^ | .41 |
| arterial hypertension | -4.2 x 10^-3^ | 1.2 x 10^-2^ | .72 | -8.1 x 10^-3^ | 5.8 x 10^-3^ | .17 | 4.1 x 10^0^ | 1.3 x 10^0^ | **.0026** |

ABI = ankle-brachial-pressure-index
